# Anaphylaxis to three humanized antibodies for severe asthma: a case study

**DOI:** 10.1186/s13223-020-00446-w

**Published:** 2020-06-10

**Authors:** Koichi Jingo, Norihiro Harada, Toshihiko Nishioki, Masahiro Torasawa, Tomoko Yamada, Tetsuhiko Asao, Haruhi Takagi, Tomohito Takeshige, Jun Ito, Kazuhisa Takahashi

**Affiliations:** 1 Department of Respiratory Medicine, Juntendo University Faculty of Medicine and Graduate School of Medicine, 3-1-3 Hongo, Bunkyo-ku, Tokyo, 113-8431 Japan; 2 Research Institute for Diseases of Old Ages, Juntendo University Faculty of Medicine and Graduate School of Medicine, Tokyo, Japan; 3 Clinical Research Center for Allergy and Rheumatology, National Hospital Organization, Sagamihara National Hospital, Kanagawa, Japan

**Keywords:** Anaphylaxis, Humanized antibody, Benralizumab, Omalizumab, Mepolizumab

## Abstract

**Background:**

Omalizumab, mepolizumab, benralizumab, and dupilumab are the currently available biologics used to treat asthma in Japan. Anaphylaxis following treatment with mepolizumab or benralizumab is considered rare.

**Case presentation:**

We report the case of a 35-year-old woman with severe asthma, who experienced anaphylaxis following the administration of benralizumab, mepolizumab, and omalizumab, separately. The therapy with biologics was chosen to avoid the repeated use of systemic corticosteroids for asthma exacerbations. The mechanisms underlying anaphylaxis caused by these three biologics remain unclear. The patient’s asthma symptoms and lung function improved after treatment with bronchial thermoplasty.

**Conclusions:**

To our knowledge, this is the first report of an asthmatic patient developing anaphylaxis after commencement of benralizumab, mepolizumab, and omalizumab therapy. These three biologics should be administered carefully, and patients should be monitored for anaphylaxis.

## Background

In Japan, in addition to the standard daily controller medication, four biologics are currently available for severe uncontrolled asthma: omalizumab (anti-immunoglobulin E (anti-IgE) monoclonal antibody), mepolizumab (anti-interleukin (anti-IL)-5 monoclonal antibody), benralizumab (anti-IL-5 receptor α (IL-5Rα) monoclonal antibody), and dupilumab (anti-IL-4 receptor α (IL-4Rα) monoclonal antibody). The use of these biologics or bronchial thermoplasty should be considered based on appropriate indications and availability to avoid frequent use of systemic corticosteroids. The use of corticosteroids is associated with complications, including osteoporosis, fractures, increased susceptibility to infections, stroke, hypertension, obesity, type 2 diabetes, cataracts, gastrointestinal ulcers, and thinning of the skin [1–3]. In patients with severe asthma, these complications increase significantly in a dose-dependent manner upon treatment with systemic corticosteroids [1].

Patients with severe asthma who have been treated for at least 6 months with a daily dose of 5 mg or more of prednisone have a significantly higher risk of developing corticosteroid-related complications than patients who have not received this treatment [1]. Moreover, exposure to four or more intermittent courses of systemic corticosteroids is associated with significantly higher odds of complications [3].

Where appropriate, the Global Asthma Initiative (GINA) guidelines for severe and difficult-to-treat asthma support the inclusion of an add-on type 2 targeted biologic for patients with exacerbations and eosinophilic and/or allergic biomarkers, despite their use of high-dose inhaled corticosteroids (ICS) and long-acting beta-agonist (LABA) therapy with or without systemic corticosteroids (daily oral corticosteroid (OCS)) [4]. Add-on omalizumab treatment can be considered for patients aged 6 years and older with moderate or severe allergic asthma that remains uncontrolled with GINA step 4 treatment. Add-on mepolizumab, benralizumab, or dupilumab treatment may also be considered for patients aged 12 years and older with severe eosinophilic or type 2 asthma that remains uncontrolled with GINA step 4 treatment [4–10]. As these biologics have a certain homology and affinity toward human tissues, they are generally well-tolerated in humans. Omalizumab was the first approved humanized antibody for the treatment of asthma in Japan. Although generally quite well-tolerated, there are many reports regarding potential adverse effects, including rare anaphylactic reactions [11–17]. However, anaphylactic reactions associated with mepolizumab or benralizumab treatment have rarely been reported [18–20]. To our knowledge, this is the first case of anaphylaxis following the administration of benralizumab, mepolizumab, and omalizumab, separately, in a patient with asthma.

### Case presentation

A 35-year-old woman was initially diagnosed with asthma at the age of 16 years. The patient presented to our hospital 3 years ago with a 1-month history of an asthma exacerbation. The diagnosis of asthma was confirmed based on a clinical history of episodic symptoms with airflow limitation and variation in pulmonary function evaluated by forced expiratory volume in 1 s (FEV_1_), in accordance with the Asthma Prevention and Management Guideline 2015, Japan [21]. She required a daily dose of 1000 μg fluticasone propionate (ICS), 40 μg formoterol (LABA), 5 μg tiotropium (muscarinic antagonist), 10 mg montelukast (leukotriene receptor antagonist), 400 mg theophylline (methylxanthine), and 5 mg oral prednisolone to manage her severe persistent asthma. Despite the high dose of ICS-LABA with daily OCS, her asthma control test (ACT) score was less than 20, which is defined as uncontrolled asthma, and she needed an increase in systemic corticosteroids for a month. She met the criteria of the European Respiratory Society (ERS) and the American Thoracic Society (ATS) for the diagnosis of severe/refractory asthma [22]. Her peripheral blood eosinophil count was 117 cells/μL, total immunoglobulin E (IgE) level was 639 IU/mL, and specific IgE against house dust mites (*Dermatophagoides farinae*) was positive (chemiluminescence enzyme immunoassay class 4). Her repeated exacerbations of asthma, blood eosinophil count, and serum IgE level under maintenance treatment with daily OCS made us consider starting her on anti-IL-5 and anti-IgE treatment. To avoid the excessive use of systemic corticosteroids, she agreed to receive her first subcutaneous administration of 100 mg mepolizumab 2 years ago. She opted for mepolizumab treatment every 4 weeks over omalizumab treatment every 2 weeks, from the perspective of work convenience (benralizumab was not yet available at that point). Her FEV_1_ and ACT scores before treatment with mepolizumab were 1.99 L and 8 points, respectively, which increased to 2.61 L and 24 points, respectively, 3 months after treatment with mepolizumab. Her clinical symptoms of uncontrolled asthma disappeared; hence, the dose of oral corticosteroids was tapered off and discontinued completely after 9 months of treatment with mepolizumab. However, she occasionally experienced asthma exacerbations, which required systemic corticosteroid administration, although mepolizumab was partially effective in alleviating her symptoms for 1.5 years. To avoid the repeated exacerbations of asthma, she expressed an interest in trying a different therapy after the 19th administration of mepolizumab and agreed to receive 30 mg benralizumab subcutaneously. Within 10 min of administration, she developed a skin rash, throat discomfort, dyspnea, and wheezing without any gastrointestinal symptoms. Her blood pressure was 127/68 mmHg, pulse rate 65 beats/min, and oxygen saturation (SpO_2_) 98%. These symptoms resolved prior to the administration of any treatment, however, she was subsequently given intravenous antihistamines and corticosteroids as per our institute’s protocol. She did not develop a late-phase anaphylactic reaction.

Two weeks after this episode, she again developed marked symptoms of an exacerbation of severe asthma. Her symptoms did not improve despite the use of systemic corticosteroids. Owing to her persistent symptoms of asthma, a severe response to benralizumab, and a desire to avoid oral corticosteroid treatment, she requested the resumption of mepolizumab treatment. Although bronchial thermoplasty was suggested as the next treatment option, she preferentially chose treatment with biologics over bronchial thermoplasty, since hospitalization for the latter procedure would impair work life. It also came to light that she had a history of allergic contact dermatitis, which was possibly triggered by polysorbate, an excipient in a milky lotion that she had used. Since benralizumab and mepolizumab formulations contain polysorbate (Table 1), a skin-prick test (SPT) to evaluate her sensitivity to polysorbate 20 was performed. After her result was confirmed to be negative (Table 2A), she received her 20th subcutaneous administration of 100 mg mepolizumab. However, 2 h after administration, an anaphylactic reaction was observed; she developed skin rash, throat discomfort, dyspnea, and wheezing without gastrointestinal symptoms. Her blood pressure was 120/68 mmHg, pulse rate 55 beats/min, and SpO_2_ 97%. These symptoms subsided without epinephrine injection, however she was again treated with intravenous antihistamines and corticosteroids per our protocol. She again did not develop a late-phase anaphylactic reaction.

**Table 1:** Components of each biologics

Benralizumab 30 mg	
Benralizumab	30 mg
l-histidine	1.4 mg
l-histidine monohydrochloride monohydrate	2.3 mg
Trehalose monohydrate	95 mg
Polysorbate 20	0.06 mg

Mepolizumab 100 mg	
Mepolizumab	144 mg
Purified sucrose	230.4 mg
Disodium hydrogen-phosphate	10.29 mg
Polysorbate 80	0.96 mg

Omalizumab 75 mg	
Omalizumab	129.6 mg
Purified sucrose	93.1 mg
l-histidine	1.8 mg
l-histidine monohydrochloride monohydrate	1.2 mg
Polysorbate 20	0.3 mg

**Table 2:** The results of SPT and DLST

A. SPT after benralizumab treatment.	Wheal (mm)/flare (mm)
Histamine	7 × 7/22 × 22
Saline	1 × 1/1 × 1
Polysorbate 20	2 × 2/2 × 2

B. SPT after omalizumab treatment.	Wheal (mm)/flare (mm)
Benralizumab	4 × 4/4 × 4
Mepolizumab	2 × 2/2 × 2
Omalizumab	2 × 2/2 × 2

C. DLST after omalizumab treatment.	%
Benralizumab	232
Mepolizumab	78
Omalizumab	117

Abbreviations for all tables: *DLST* drug-induced lymphocyte stimulation test; *SPT* skin-prick test

Although there was an improvement in the anaphylaxis induced by the mepolizumab injection, she required hospitalization for a month owing to the exacerbation of her asthma. To reduce the dose of administered systemic corticosteroids, she requested that her therapy be changed to omalizumab. Accordingly, she was administered a subcutaneous dose of 450 mg omalizumab, while the corticosteroid therapy was tapered (intravenous administration of 4 mg betamethasone). No anaphylactic reaction was observed. After the first administration of omalizumab, she was discharged without any indicative symptoms of asthma. Two weeks later, she was re-hospitalized for her second dose of omalizumab. However, the second dose caused anaphylaxis, and she experienced itchy skin, throat discomfort, dyspnea, wheezing, and transient loss of consciousness without gastrointestinal symptoms. Her vital signs within 30 min of this episode were as follows: a drop-in blood pressure to 86/54 mmHg, a pulse rate of 80 beats/min, and an SpO_2_ value of 88%. Oxygen was administered by nasal prongs at a rate of 3 L/min. Subsequent intramuscular epinephrine injection resulted in her regaining consciousness and an immediate improvement of hypotension. Her general condition improved within 50 min of receiving epinephrine. She was also treated with an intravenous drip of 40 mg methylprednisolone and chlorpheniramine maleate (histamine H1 receptor antagonist). Six hours after omalizumab administration, she experienced a late-phase anaphylaxis, which was not observed during therapy with mepolizumab and benralizumab. She experienced itchy skin, throat discomfort, dyspnea, and transient loss of consciousness without gastrointestinal symptoms. An intramuscular injection of epinephrine and an intravenous drip of 40 mg methylprednisolone were administered; her symptoms improved immediately.

She required systemic corticosteroids (oral prednisolone 5 mg/day) for severe persistent asthma and an intermittent high dose of corticosteroids for repeated severe exacerbation of asthma for 6 months after the anaphylaxis following omalizumab administration. She was also treated with bronchial thermoplasty, and no adverse events were experienced. After the third bronchial thermoplasty, she is currently being treated without systemic corticosteroids and her condition has been stable for 4 months. Moreover, her FEV_1_ and ACT scores have increased to 2.79 L and 25 points, respectively.

### Discussion and conclusions

In this paper, we present the first reported case of anaphylaxis induced by three humanized antibodies (benralizumab, mepolizumab, and omalizumab), which are generally considered safe and indicated for use in severe asthma. Anaphylaxis is a rapid, systemic, and a potentially life-threatening immune reaction that requires immediate pharmacological intervention. Epinephrine is universally recommended as the first-line therapy for anaphylaxis to prevent a potentially fatal outcome and works best when administered at the onset of the reaction [23–26]. In addition, non-administration or delayed administration of epinephrine increases the risk of death [27, 28]. Thus, epinephrine should be administered to all patients who experience anaphylaxis. However, it has been reported that only 1 in 4 patients with severe anaphylaxis are administered epinephrine, and these statistics have remain unchanged over the past decade [29]. A possible reluctance to use epinephrine as the first-line therapy in anaphylaxis owing to unfounded fear of the drug itself may be a factor deterring its use [30]. The significant discrepancy between the recommended and actual practice suggests that there is a need to educate and advocate the use of epinephrine at the onset of clinical anaphylaxis. In the present case, the patient was neither hypotensive nor desaturated during her two episodes of anaphylaxis following treatment with benralizumab and mepolizumab. However, considering the patient’s history of acute respiratory disorder with unstable asthma, it would have been more appropriate to administer epinephrine immediately, rather than delay or not administer epinephrine, even if her symptoms improved spontaneously in those two episodes. Furthermore, her symptoms improved spontaneously without epinephrine injection, suggesting that she may not have experienced anaphylaxis. However, this should not be the reason to refrain from epinephrine therapy, and in hindsight, our patient should have been administered epinephrine immediately once anaphylaxis was suspected. In addition, there is a lack of evidence to support the efficacy of glucocorticoids and antihistamines, including H1- and H2-antihistamines, in the management of anaphylaxis [30, 31]. Therefore, given that these therapies could delay the timely administration of epinephrine, it should be emphasized to administer epinephrine as first-line therapy [30–32].

Furthermore, omalizumab, but not benralizumab and mepolizumab, induced a late-phase anaphylactic reaction, 6 h after administration. Late-phase anaphylactic reactions to allergens have been studied mainly in cutaneous models, leukocytes (eosinophils and smaller numbers of neutrophils and basophils), and monocytes recruited during late-phase reactions in the human skin [33]. Benralizumab and mepolizumab can suppress eosinophils and consequently inhibit the late-phase anaphylactic reaction. However, this hypothesis needs further investigation.

Biologics should be used cautiously, and clinicians should be aware of the possibility of anaphylactic reactions to different/multiple agents if a patient develops anaphylactic reactions to one of the biologics in use. After the first episode of anaphylaxis induced by benralizumab, bronchial thermoplasty was considered for the next treatment option. Our present patient experienced an improved quality of life, increased respiratory functionality, and fewer exacerbations of asthma after successful bronchial thermoplasty. Bronchial thermoplasty might thus be an option for asthmatic patients who do not respond well to pharmacological treatment; however, the long-term safety of bronchial thermoplasty treatment is still unclear [34, 35]. Moreover, owing to severe uncontrolled asthma in our patient despite the use of high dose of corticosteroids, we were unable to decide if bronchial thermoplasty during the initial treatment was a viable option.

Polysorbate, an additive used to increase the solubility of poorly water-soluble drugs, is one of the excipients in these three biologics responsible for hypersensitivity reactions [11–17, 36–38]. There are four types of polysorbates used as excipients: polysorbates 20, 40, 60, and 80, of which, polysorbates 20 and 80 are more common [37]. Benralizumab and omalizumab used in our clinical setting contained polysorbate 20 as an emulsion stabilizer, whereas mepolizumab contained polysorbate 80 (Table 1). The anaphylactic reaction following the first administration of benralizumab may have been due to the presence of polysorbate 20. Our patient presented with a history of allergic rhinitis, pollinosis, skin rash after a meal of raw shrimps as well as allergic contact dermatitis caused by a milky lotion, which may have contained polysorbate as an excipient. However, as shown in Table 2, her SPT results for reactions due to polysorbate 20, mepolizumab, and omalizumab were negative. Additionally, she tested negative for the drug-induced lymphocyte stimulation test (DLST) for mepolizumab and omalizumab (Table 2). However, she tested positive for the SPT and DLST for benralizumab (Table 2). These tests are commonly used for the auxiliary diagnosis of drug allergies [39–46]. However, the results of the SPT for drug allergies are drug-dependent and not always reliable, for many drugs the sensitivity and specificity have not been established [39]. Similarly, the DLST can produce false-positive or false-negative results, and the drug-allergy results greatly depend on the drug of interest [40, 41]. Therefore, there is a possibility that the biologics used in this study displayed false-positive or false-negative results in SPTs and DLSTs. Peripheral blood mononuclear cells (PBMCs) were isolated from whole blood by density-gradient centrifugation and used for the DLST. Since benralizumab interacts directly with PBMC through the IL-5 receptor α unit, interpreting these DLST results could be challenging. Further studies are required to investigate why all three biologics induced anaphylaxis in the patient, although she showed positive results for the SPT and DLST only for benralizumab.

Mast cell activation syndrome (MCAS) and mastocytosis are also possible causes of repeated episodes of anaphylaxis [47–51]. Hypotension is an important diagnostic parameter commonly manifesting in mast cell activation (MCA) and MCAS. Moreover, the diagnosis of MCAS is strongly supported by acute serum tryptase levels higher than plus 20% of the baseline [47–51]. In the present case, the lack of hypotension during anaphylaxis following benralizumab and mepolizumab administration did not make us aware of the possibility of MCA and MCAS. The patient’s baseline serum tryptase levels were 3.1, 2.5, and 2.9 μg/L, before treatment with mepolizumab, benralizumab, and bronchial thermoplasty, respectively; her acute serum tryptase levels were not measured after the anaphylactic reaction. When anaphylactic reactions are severe and recurrent, the diagnoses of MCA and MCAS must be considered [47–51]. Therefore, the lack of this data is a limitation of our case presentation.

In conclusion, to the best of our knowledge, this is the first report of an asthmatic patient developing anaphylaxis following the administration of three commonly used biologics to treat asthma—benralizumab, mepolizumab, and omalizumab. Since the reasons for the anaphylaxis are unclear, these biologics should be administered carefully in clinical settings.

## Data Availability

Data sharing is not applicable to this article as no datasets were generated or analyzed during the current study.

## Abbreviations

ACT: Asthma control test
BT: Bronchial thermoplasty
DLST: Drug-induced lymphocyte stimulation test
FEV_1_: Forced expiratory volume in 1 s
GINA: Global Asthma Initiative
ICS: Inhaled corticosteroid
IgE: Immunoglobulin E
IL: Interleukin
LABA: Long-acting beta-agonist
MCA: Mast cell activation
MCAS: Mast cell activation syndrome
OCS: Oral corticosteroid
PBMC: Peripheral blood mononuclear cells
SpO_2_: Oxygen saturation
SPT: Skin-prick test
